# KUS121, a valosin-containing protein modulator, attenuates ischemic stroke via preventing ATP depletion

**DOI:** 10.1038/s41598-019-47993-w

**Published:** 2019-08-08

**Authors:** Hisanori Kinoshita, Takakuni Maki, Ken Yasuda, Natsue Kishida, Norio Sasaoka, Yasushi Takagi, Akira Kakizuka, Ryosuke Takahashi

**Affiliations:** 1 Department of Neurology, Kyoto University Graduate School of Medicine, 54 Shogoin-Kawahara-cho, Sakyo-ku, Kyoto, 606-8507 Japan; 2 Department of Neurosurgery, Kyoto University Graduate School of Medicine, Kyoto, Japan; 3 Laboratory of Functional Biology, Kyoto University Graduate School of Biostudies, Kyoto, Japan

**Keywords:** Cellular neuroscience, Stroke

## Abstract

Reduced adenosine triphosphate (ATP) levels in ischemic stroke constitute an upstream contributor to neuronal cell death. We have recently created a small chemical, named Kyoto University Substance 121 (KUS121), which can reduce cellular ATP consumption. In this study, we examined whether KUS121 has neuroprotective effects in rodent cerebral ischemia models. We evaluated cell viability and ATP levels *in vitro* after oxygen glucose deprivation (OGD) in rat cortical primary neuronal cultures incubated with or without KUS121. We found that KUS121 protected neurons from cell death under OGD by preventing ATP depletion. We also used *in vivo* ischemic stroke models of transient distal middle cerebral artery occlusion in C57BL/6 and B-17 mice. Administration of KUS121 in these models improved functional deficits and reduced brain infarction volume after transient focal cerebral ischemia in both C57BL/6 and B-17 mice. These results indicate that KUS121 could be a novel type of neuroprotective drug for ischemic stroke.

## Introduction

Stroke is one of the most common causes of death worldwide^1^. In addition to antithrombotic therapy^2^, intravenous thrombolysis with alteplase and mechanical thrombectomy have been shown to be very effective from clinical evaluations, albeit in a limited number of patients with ischemic stroke in the acute phase^3,4^. Therefore, the main target of acute stroke treatment is to salvage the ischemic penumbra, which is a hypoperfused and non-functional, yet still viable, area surrounding the ischemic core^5^. However, numerous neuroprotective drugs that modulate key pathogenic factors after cerebral ischemia, including excitotoxicity, oxidative and nitrosative stress, or inflammation, have failed to show significant benefits in the clinical treatment of acute ischemic stroke^6^. Hence, there is an urgent need to explore new neuroprotective agents or strategies for acute ischemic stroke.

Acute brain ischemia is characterized by an abrupt decrease in cerebral blood flow and insufficiency to supply an adequate amount of oxygen and nutrients. Adenosine triphosphate (ATP) levels rapidly decrease in the ischemic lesions during the first 5 min after the onset of cerebral ischemia. The resultant ATP shortage and energy failure lead to downstream cascades, including excitatory neurotoxicity, calcium overload, ionic imbalance, oxidative and nitrosative stress, endoplasmic reticulum (ER) stress, and further inflammatory and metabolic disruption of the affected ischemic tissue^6^. Thus, drugs that prevent ATP depletion early after the stroke are expected to alleviate the ischemic brain damage.

**Figure 1:**
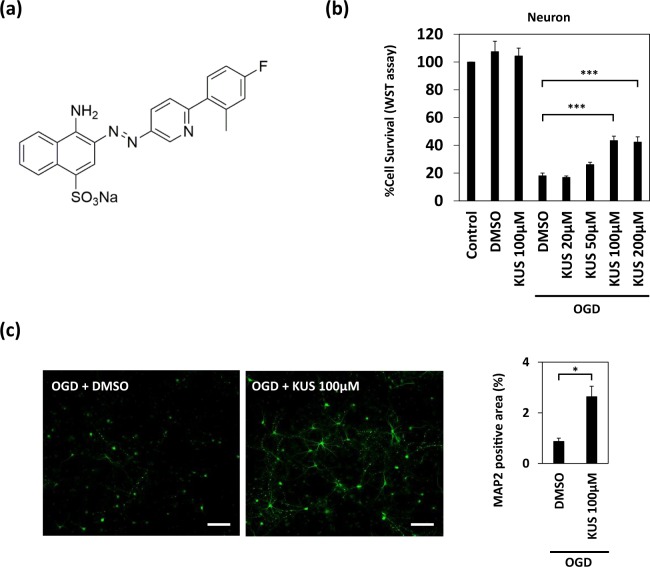
KUS121 protects primary cortical neurons under oxygen glucose deprivation (OGD). (**a**) The chemical structure of KUS121, an ATPase inhibitor of valosin-containing protein (VCP). (**b**) Cell viability measured by WST (water-soluble tetrazolium salt) assay. Primary cortical neurons from the rats were exposed to vehicle (dimethyl sulfoxide, DMSO) or KUS121 under control conditions or under OGD for 2 h (n = 4; ***p < 0.001). (**c**) Left: MAP2 stained rat primary cortical neurons after OGD. Primary neurons were exposed to OGD for 1.5 h in the presence of vehicle (DMSO) or 100 μM KUS121. Scale bar: 100 μm. Right: Graph showing the ratio of MAP2-positive area to the total image area (n = 4; *p < 0.05).

Kyoto University Substance 121 (KUS121), a newly generated compound, is an inhibitor of ATPase activities of valosin-containing protein (VCP), the most abundant soluble ATPase in mammalian cells, including neurons (Fig. 1a)^7^. VCP is a ubiquitously expressed AAA (ATPase Associated with diverse cellular Activities)-type ATPase, considered as a major factor in neurodegeneration^8^. In addition to its ATPase activity, VCP is thought to be involved in many cellular functions, including ER-associated and proteasome-mediated protein degradation^8^. KUS121 was shown to specifically inhibit the ATPase activity of VCP in pathological conditions, without apparently inhibiting its other cellular functions^7^. KUS121 has already been demonstrated to exert significant neuroprotective effects in animal models of several diseases, including Parkinson’s disease, retinal ischemic injury, glaucoma, and retinitis pigmentosa^7,9–12^. Nonetheless, its efficacies in brain ischemia models have not been investigated.

In this study, we examined whether KUS121 is effective in *in vitro* and *in vivo* cerebral ischemia models. We used oxygen glucose deprivation (OGD) in primary neuron and oligodendrocyte cultures as an *in vitro* model of ischemic stroke^13^. To test KUS121 effects *in vivo*, we used a mouse model of transient distal middle cerebral artery (MCA) occlusion (MCAO)^14,15^. To confirm reproducibility, we performed the ischemic experiment in two mouse strains.

## Results

### KUS121 protects cortical neurons *in vitro* under OGD by preventing ATP depletion

To assess the protective effects of KUS121 on neurons under ischemic conditions, we assessed cell viability after OGD in both the presence and absence of KUS121. OGD was performed in rat cortical primary neuronal cultures for 2 h, with a subsequent recovery at 21% O_2_ and in a glucose-containing media for another 22 h. Control neurons were kept at 21% O_2_ in glucose-containing media for 24 h. Exposure to OGD induced cell death in primary cortical neurons. However, simultaneous incubation with 100 or 200 μM KUS121 significantly improved neuronal viability after OGD; the viability was 18.1 ± 1.9% in vehicle (dimethyl sulfoxide, DMSO)-treated neurons, 43.5 ± 3.1% in neurons treated with 100 μM KUS121, and 42.4 ± 3.7% in those treated with 200 μM KUS121 (p < 0.001; Fig. 1b, Dunnett’s test). Furthermore, 100 μM KUS121 significantly increased the microtubule-associated protein 2 (MAP2, a well-established marker for neurons)-positive area compared to vehicle (DMSO, 0.87 ± 0.13%; 100 μM KUS121, 2.64 ± 0.41%; p < 0.05; Fig. 1c, Student’s t test). DMSO or KUS121 without OGD had no effect on neuronal viability (control: 100 ± 0.0%; vehicle (DMSO): 107.5 ± 7.5%, p = 0.60; KUS121: 104.4 ± 5.6%, p = 0.94; Fig. 1b, Dunnett’s test).

**Figure 2:**
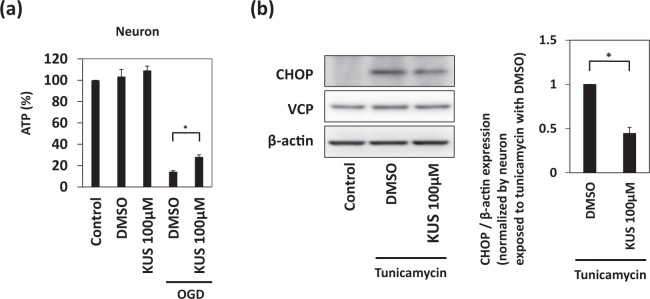
KUS121 prevents ATP depletion and endoplasmic reticulum (ER) stress. (**a**) Measurement of ATP concentration. Primary cortical neurons were exposed to vehicle (dimethyl sulfoxide, DMSO) or 100 μM KUS121 under control conditions or under OGD for 1.5 h. Then, ATP was measured (n = 6; *p < 0.05). (**b**) Left: Western blot analyses on CCAAT/enhancer-binding protein homologous protein (CHOP) and valosin-containing protein (VCP). Primary cortical neurons were treated with 0.25 μg/mL tunicamycin for 6 h with vehicle (DMSO) or 100 μM KUS121 and then subjected to western blot analyses. Right: Graph showing the ratio of CHOP to β-actin expression levels (n = 4; *p < 0.05).

Since KUS121 reportedly prevents ATP depletion in pathological conditions^7,9–12^, we then examined whether KUS121 shows similar effects in primary cortical neurons under OGD. Cellular ATP levels were measured by a luciferase-based assay after 1.5 h of OGD. Decreased ATP levels after OGD were rescued by KUS121 treatment (DMSO with OGD: 14.3 ± 0.9%; KUS121 with OGD: 28.2 ± 1.9%; p < 0.05.; Fig. 2a, Dunnett’s test). Under normoxic conditions, DMSO or KUS121 had no effect on ATP levels (control: 100 ± 0.0%; DMSO: 103.5 ± 6.7%, p = 0.91; KUS121: 109.1 ± 4.0%, p = 0.29; Fig. 2a, Dunnett’s test). These data suggest that KUS121 protects primary cortical neurons under OGD by preventing ATP depletion. The purity of the culture was validated by using immunoblot analysis and immunocytochemistry. (Supplementary Fig. S1a,b).

### KUS121 protects primary cortical neurons under ER stress

Since ER stress is induced after ischemia^16^, we additionally tested the protective effect of KUS121 in primary cortical neurons treated with tunicamycin, instead of OGD (see discussion). Tunicamycin is known to cause ER stress^17^. CCAAT/enhancer-binding protein homologous protein (CHOP) is a core mediator of ER stress-induced cell death and is upregulated during ER stress^18,19^. Cortical neurons were exposed to 0.25 μg/mL tunicamycin for 6 h. In these conditions, KUS121 suppressed the expression of CHOP (DMSO: 1.0 ± 0.0; KUS121: 0.45 ± 0.07; p < 0.05; Fig. 2b, Student’s t test).

### Treatment with KUS121 improves functional deficits and reduces brain infarction volume

**Figure 3:**
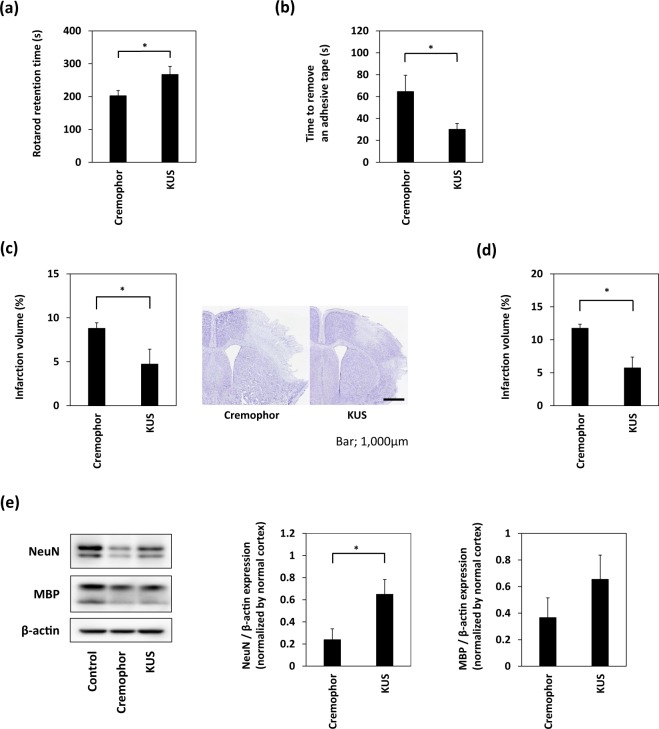
Treatment with KUS121 improves functional deficits and reduces brain infarction volume. (**a**) Rotarod retention time and (**b**) the time to remove an adhesive tape in C57BL/6 mice subjected to transient occlusion of the distal portion of left middle cerebral artery (MCA). Neurological function was measured by the accelerating rotarod apparatus and adhesive removal test 24 h after occlusion of the distal MCA (n = 16; *p < 0.05). (**c**) Quantification of the infarction volume was calculated by Nissl staining and representative Nissl stained brain sections of C57BL/6 mice (Scale bar: 1,000 μm; vehicle, n = 5; KUS121, n = 4; *p < 0.05). (**d**) Quantification of the infarction volume in B-17 mice measured 24 h after transient occlusion of the distal MCA and calculated by Nissl staining (vehicle, n = 4; KUS121, n = 5; *p < 0.05). (**e**) Western blot analyses of cerebral cortex lysates for Neuronal Nuclei (NeuN) and myelin basic protein (MBP). The cerebral cortex of B-17 mice was collected 24 h after transient occlusion of the distal MCA. The ratio of NeuN or MBP to β-actin expression levels was calculated (n = 5; *p < 0.05).

To evaluate the effect of KUS121 on cerebral ischemia, transient focal cerebral ischemia was induced in C57BL/6 mice. Vehicle (5% Cremophor) or 100 mg/kg KUS121 was intravenously administered via the tail vein immediately after the occlusion of the distal portion of the left MCA. Intraperitoneal administration was added at 50 mg/kg after the reperfusion. Twenty four hours after the occlusion, KUS121 treatment significantly prolonged the retention time on the rotarod, compared with vehicle treatment (vehicle: 202.5 ± 15.7 s, KUS121: 267.1 ± 24.5 s, p < 0.05; Fig. 3a, Student’s t test), and significantly reduced the time to remove the tape in the adhesive removal test (vehicle: 64.6 ± 14.3 s, KUS121: 30.1 ± 5.2 s, p < 0.05; Fig. 3b, Student’s t test). In addition, KUS121 treatment significantly reduced the infarction volume (vehicle: 8.8 ± 0.7%, KUS121: 4.7 ± 1.7%, p < 0.05; Fig. 3c, Student’s t test).

The neuroprotective effect of KUS121 was also confirmed in a CB-17 mouse model of ischemic stroke. Intravenous administration of 100 mg/kg KUS121 immediately before ischemia with 50 mg/kg intraperitoneal administration after reperfusion reduced the infarction volume (vehicle: 11.8 ± 0.6%, KUS121: 5.7 ± 1.6%, p < 0.05; Fig. 3d, Student’s t test). Furthermore, western blot analysis of cerebral cortex lysates revealed that KUS121 treatment rescued the Neuronal Nuclei (NeuN) and Neurofilament Heavy polypeptide (NF-H) expression levels, compared with vehicle treatment (NeuN; vehicle: 0.24 ± 0.10, KUS121: 0.65 ± 0.13, p < 0.05; Fig. 3e, Student’s t test and NF-H; vehicle: 0.28 ± 0.11, KUS121: 0.82 ± 0.13, p < 0.05; Supplementary Fig. S1c, Student’s t test).

### KUS121 has no effect on oligodendrocyte viability under ischemia

**Figure 4:**
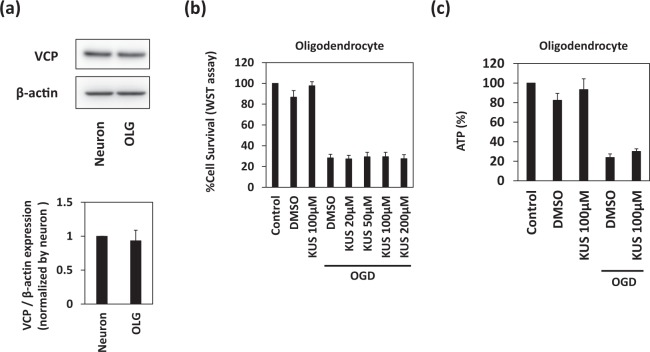
KUS121 has no effect on the viability of oligodendrocytes under ischemia. (**a**) Western blot analyses of rat primary cortical neuron and oligodendrocyte lysates for VCP. The ratio of VCP to β-actin expression levels was calculated (n = 4). (**b**) Cell viability measured by the WST (water-soluble tetrazolium salt) assay. Rat primary oligodendrocytes were exposed to vehicle (dimethyl sulfoxide, DMSO) or KUS121 under control conditions or under OGD for 12 h (n = 4). (**c**) Measurement of ATP concentration in primary cortical oligodendrocytes exposed to vehicle (DMSO) or 100 μM KUS121 under control or OGD conditions (n = 5).

To assess the protective effects of KUS121 on oligodendrocytes under ischemic conditions, we performed cell viability assays after OGD in oligodendrocyte cultures treated with and without KUS121. OGD was performed in rat primary oligodendrocytes for 12 h with subsequent recovery at 21% O_2_ and glucose-containing media for another 12 h. Control oligodendrocytes were kept at 21% O_2_ in glucose-containing media for 24 h. VCP expression levels were similar in primary cortical neurons and oligodendrocytes (neurons: 1.0 ± 0.0%, oligodendrocytes: 0.93 ± 0.15%, p = 0.56; Fig. 4a, Student’s t test); however, the addition of KUS121 did not significantly improve oligodendrocyte viability after OGD, as in the case of primary cortical neurons (Fig. 4b, Dunnett’s test). No significant difference in cellular ATP levels was observed after OGD between cells treated with or without KUS121 (Fig. 4c, Dunnett’s test). The purity of the culture was validated by using immunoblot analysis and immunocytochemistry. (Supplementary Fig. S1a,b).

In addition, western blot analysis of cerebral cortex lysates from CB-17 mice showed that myelin basic protein (MBP) and Glutathione S-transferase pi (GST-π) expression levels were not significantly preserved by KUS121 treatment (MBP; vehicle: 0.37 ± 0.15, KUS121: 0.65 ± 0.18, p = 0.20; Fig. 3e, Student’s t test and GST-π; vehicle: 0.53 ± 0.06, KUS121: 0.58 ± 0.08, p = 0.64; Supplementary Fig. S1c, Student’s t test).

## Discussion

KUS121 was developed as a specific inhibitor of the ATPase activity of VCP, the most abundant soluble ATPase in essentially all types of cells. In our previous studies, KUS121 prevented cellular ATP depletion in cultured cells in several *in vitro* pathological conditions, including glucose deprivations and mitochondrial respiratory chain inhibitions^7,9^. Furthermore, KUS121 improved disease phenotypes in several mouse models of retinal diseases, including retinal ischemic injury, glaucoma, and retinitis pigmentosa, by inhibiting neuronal cell death^7,9–11^. The efficiency of KUS121 was also observed in a mouse model of Parkinson’s disease generated by 1-methyl-4-phenyl-1,2,3,6-tetrahydropyridine^12^. Administration of KUS121 improved functional outcomes and increased the number of dopaminergic neurons by mitigating α-synuclein accumulation and ATP depletion^12^. Our current study demonstrates for the first time that KUS121 also exerts neuroprotective effects under cerebral ischemia. KUS121 protected rat primary cultured cortical neurons under OGD by preventing ATP depletion. Furthermore, administration of KUS121 improved functional deficits and reduced brain infarction volume in mice after transient focal cerebral ischemia.

ATP depletion is the earliest consequence of the reduced blood supply in ischemic stroke^20^. Continuation of ATP consumption, despite its minimal synthesis, leads to acidosis and loss of ionic homeostasis^21^. As a consequence, various pathophysiological events take place leading to neuronal cell death^20,22^, including ER stress. This was demonstrated by both *in vitro* and *in vivo* experiments^16^. For example, the ER stress-associated factor CHOP is increased after ischemia, while CHOP-deficient mice show decreased neuronal loss after ischemia. Moreover, disrupted ionic homeostasis following ATP depletion triggers the release of glutamate, an excitatory neurotransmitter, while excessive calcium influx through N-methyl-D-aspartic acid (NMDA) receptors leads to excitatory neuronal cell death^23,24^. Although there are many expected neuroprotective agents for ischemic stroke, they do not directly affect ATP depletion^25^. For example, antagonists of NMDA receptors, i.e., ligand-gated ionotropic glutamate receptors^26^, reduce calcium influx into neurons^27^. In addition, several reports have shown the efficiency of anti-oxidants and anti-inflammatory agents on cerebral ischemia^28,29^, as ATP depletion and excessive calcium in ischemic cells stimulate production of reactive oxygen species^21^. Moreover, it is known that oxidative stress and inflammation are closely related to the pathophysiological processes of cerebral ischemia^28,29^, while inflammatory cells, including resident microglia, peripheral neutrophils, and T cells, are time-dependently activated and recruited^20^. However, these constitute relatively downstream pathophysiological events of ischemic stroke^20,21^. The advantage of KUS121 is that it affects ATP depletion, which is the most upstream and core pathological event of ischemic stroke^20^.

This study, however, has several limitations. First, ER stress could not be assessed under ischemic conditions. This is because primary cortical neurons are so vulnerable to OGD (Supplementary Fig. S2a) that the long hours required to induce CHOP or GRP78 protein level caused almost all cells to die^30^. Therefore, we used tunicamycin to induce ER stress, as an alternative method. Second, we did not assess the long-term effect of KUS121 on ischemic stroke, as we analyzed the infarct volume and functional deficits 24 h after ischemia induction in mice. This is because functional deficits in mice with distal MCAO did not persist sufficiently until 7 days after stroke (Supplementary Fig. S2b,c). Future studies are needed to determine the long-term effects of KUS121. Third, although we found VCP expression to be similar between rat primary oligodendrocytes and cortical neurons, we could not identify any significant effects of KUS121 either on ATP levels or on oligodendrocyte viability. Although the precise mechanism for this difference is unclear, it may occur through drug influx, metabolism, or efflux. For example, isoforms of multidrug-resistant proteins that are associated with drug efflux are differentially expressed in neurons and glial cells^31^. In addition, isoforms of cytochrome P450, a metabolic enzyme, are also differentially expressed in these two cell types^32,33^. Further investigation is warranted to elucidate the detailed mechanisms that cause the apparently different effects of KUS121 on neurons and oligodendrocytes. Finally, we did not consider the detrimental role of ATP under cerebral ischemia. Following ischemia, there is a rapid accumulation of extracellular ATP^34^, which was shown to have neurotoxic functions^35,36^. Although our *in vivo* study showed KUS121 had beneficial effects during ischemia, extracellular ATP levels were not assessed. In addition, given the regional difference of ATP levels after ischemic stroke, temporal profiles of intra- and extracellular ATP levels in the ischemic core and penumbra after MCAO with or without KUS121 should be evaluated in the future studies.

In summary, KUS121 improves the functional outcomes and attenuates the infarct volume after ischemic stroke in mice. The neuroprotective effect of KUS121 is mediated by prevention of ATP depletion under ischemia, as shown by our *in vitro* experiments. The results of the present study indicate that KUS121 may be a novel promising agent for the treatment of ischemic stroke.

## Materials and Methods

### Primary cell cultures

#### Primary oligodendrocyte cell culture

Oligodendrocytes were prepared as previously described^37^. Briefly, cerebral cortices from 1–2-day-old Sprague Dawley rats (Shimizu Laboratory Supplies) were dissected, minced, and digested. Dissociated cells were plated in poly-D-lysine-coated 75-cm^2^ flasks and maintained in Dulbecco’s Modified Eagle’s Medium (DMEM) containing 20% heat-inactivated fetal bovine serum and 1% penicillin/streptomycin. After the cells reached confluency (~10 days), the flasks were shaken for 1 h on an orbital shaker (220 rpm) at 37 °C, to remove microglia. They were then changed to new medium and shaken overnight (~24 h). The medium was collected and plated on non-coated tissue culture dishes for 1 h at 37 °C, to eliminate contaminating astrocytes and microglia. The non-adherent cells, i.e., oligodendrocyte precursor cells, were collected and re-plated in Neurobasal (NB) medium containing 2 mM glutamine, 1% penicillin/streptomycin, 10 ng/mL platelet-derived growth factor AA, 10 ng/mL fibroblast growth factor-2, and 2% B27 supplement onto poly-DL-ornithine-coated plates. Three to 5 days after plating, the culture medium was switched to DMEM containing 1% penicillin/streptomycin, 10 ng/mL ciliary neurotrophic factor, 15 nM thyroid hormone T3, and 2% B27 supplement, to differentiate oligodendrocyte precursor cells to oligodendrocytes.

#### Primary neuronal cell culture

Cortical neuronal cultures were prepared from 17-day-old Sprague Dawley rat embryos (Shimizu Laboratory Supplies), as described previously^38^. Briefly, cortices were dissected and dissociated. Cells were plated on dishes coated with poly-D-lysine in DMEM, containing 5% heat-inactivated fetal bovine serum, and 1% penicillin/streptomycin, at a density of 200,000–250,000 cells/cm^2^. 24 h after seeding, the medium was changed to NB medium containing 0.5 mM glutamine, 1% penicillin/streptomycin, and 2% B27 supplement. Cultured neurons were used for experiments 14 days after seeding.

### Oxygen glucose deprivation

The medium was replaced with DMEM without glucose. The cells were then placed into a sealed Anaero container with an Anaero Pack (Mitsubishi Gas Chemical Company) for 1.5–12 h. Following OGD, cells were switched to normal medium and returned to a normoxic 5% CO2 incubator.

### Cell viability test

Cell viability was assessed using the Cell Counting Kit-8 (CCK-8) assay (Dojindo Laboratories), which is based on the conversion of a water-soluble tetrazolium salt [2-(2-methoxy-4-nitrophenyl)-3-(4-nitrophenyl)-5-(2,4-disulfophenyl)-2H-tetrazolium, monosodium salt] to a water-soluble formazan dye upon reduction by dehydrogenases in the presence of an electron carrier. The cells were incubated with 10% CCK-8 solution for 1–2 h at 37 °C. Then, the absorbance of the culture medium was measured using a microplate reader at a test wavelength of 450 nm and a reference wavelength of 630 nm.

### Immunocytochemistry

The cells were washed twice with phosphate buffered saline (PBS), followed by 4% paraformaldehyde (PFA) for 15 min. After being further washed twice with PBS, they were incubated in PBS/0.1%Tween for 10 min and blocked with 3%BSA/PBS for 1 h at room temperature. The cells were incubated with a primary antibody against MAP2 (1:1000; Sigma Aldrich, M1406) or MBP (1:500; Thermo Fisher, MA1-10837) at 4 °C overnight. Subsequently, after being washed with PBS, cells were incubated with a secondary antibody conjugated with Alexa Fluor 488 (1:500; Thermo Fisher, A28175) or Alexa Fluor 594 (1:500; Thermo Fisher, A11005) for 1 h at room temperature. Finally, nuclei were counterstained with 4′, 6′-diamidino-2-phenylindole (Vector Laboratories, H-1200). Images were taken with a fluorescence microscope (KEYENCE BZ-X 710) by an investigator who was blinded to the experimental groups. The images were exported into ImageJ software (National Institutes of Health) in TIFF format for processing. MAP2-positive area was automatically delineated by using the “auto setting threshold” (default method). The ratio of MAP2-positive area to the total image area was calculated.

### ATP assay

ATP levels in cell lysates were measured using a luciferase chemiluminescence-based ATP assay for cells (Toyo Ink), in accordance with the manufacturer’s protocol. Briefly, 100 μL of the lysis solution, provided by the manufacturer, was added to each well of 96-well plates. After incubating for 5 min at room temperature, the luminescence of an aliquot of the solution was measured in a luminometer.

### Western blotting

Cells were collected and lysed in RIPA buffer (20 mM HEPES-KOH pH 7.4, 150 mM NaCl, 2 mM EDTA, 1% Nonidet-P40, 1% sodium deoxycholate) containing 10% 2-mercaptoethanol (Nacalai tesque) and 1% protease inhibitor (Nacalai tesque). Dissected brain samples were also homogenized in the above buffer. Samples were sonicated and centrifuged at 4 °C and 15,000 rpm for 15 min, and the supernatant was collected. Proteins (15–20 μg per lane) were loaded, separated by 5–20% SDS-PAGE, and transferred to polyvinylidene fluoride membranes (Millipore). The following primary antibodies were used: β-actin (1:5000; Sigma Aldrich, A5441), CHOP (1:1000; Cell Signaling Technology, L63F7), GST-π (1:500; MBL, 312), MBP (1:1000; Thermo Fisher, MA1-10837), NeuN (1:2000; Merck Millipore, ABN78), NF-H (1:500; BioLegend, 801701) and VCP (1:500; ABGENT, AP6920b). Horseradish peroxidase-labeled secondary antibodies (Santa Cruz Biotechnology) were used for visualization by enhanced chemiluminescence (Nacalai tesque). The western blot images were captured using an Amersham Imager 600 (GE Health Care) and quantified with ImageJ software.

### Mice

Adult male C57BL/6 (C57BL/6NJcl) and CB-17 (CB-17/lcr-+/+Jcl) mice (6-7-weeks-old) were purchased from Shimizu Laboratory supplies and Clea Japan, respectively. Animals were allowed access to food and tap water *ad libitum*.

### Stroke surgery

In CB-17 mice, distal MCAO was performed as previously described, with minor modifications^14^. In brief, general anesthesia was induced and maintained by inhalation of 4% and 1.5% isoflurane (Pfizer), respectively. Mice were placed in a lateral position, and a skin incision was made between the left eyeball and left external auditory canal. The left salivary gland and part of the temporalis muscle were resected to allow visualization of the MCA through the cranial bone. A burr hole was made in the cranial bone. Then, the MCA was isolated and transiently occluded with a monofilament 6-0 nylon suture (Alfresa Pharma, HR1206NA45-KF2). After occlusion for 22 min, MCA blood flow was restored by removal of the nylon suture. During surgery, rectal temperature was monitored and controlled at 36.0–37.2 °C by a feedback-regulated heating pad.

In C57BL/6 mice, distal MCAO with hypoxia was performed as previously described with some modifications^15^. After occlusion of the distal MCA by a nylon suture, mice were placed in a large chamber containing 10% oxygen and 90% nitrogen. After 30 min of hypoxia, mice were returned to normoxic conditions, and the nylon suture was removed.

### Evaluation of stroke volume

Twenty-four hours after ischemia, mice were deeply anesthetized and perfused transcardially with PBS and 4% PFA. The brains were extracted, fixed in 4% PFA for 48 h, and were further cryoprotected in 20% sucrose, until they sunk to the bottom of the vial. For Nissl staining, the brains were cut serially on a cryostat in 20-μm thick sections, every 600 μm, and collected on slides (anterior-posterior +2.0, +1.4, +0.8, +0.2, −0.4, −1.0, and −1.6 mm relative to Bregma). The sections were incubated in cresyl violet solution for 15 min and dehydrated in 70% methanol for 5 sec. Afterwards, the slides were covered with mounting medium. Images were taken with a fluorescence microscope (KEYENCE BZ-X 710) by an investigator who was blinded to the experimental groups. The images were exported into ImageJ software in TIFF format. The area of the ipsilateral hemisphere and infarct on each section were measured using ImageJ software. Measurements were multiplied by the distance between sections (600 μm) and then summed over the entire brain to yield the volume measurements.

### Assessment of functional deficits in mice

Neurobehavioral outcomes were examined through the accelerating rotarod test and the adhesive removal test. Based on our previous studies and pilot data, appropriate sample size was calculated as follows: accelerating rotarod test; α = 0.05, β = 0.2, δ = 60, σ = 55 and adhesive removal test; α = 0.05, β = 0.2, δ = 32, σ = 30. Groups of at least 14 mice were required to achieve appropriate power for rotarod analysis, and up to 15 mice per group for adhesive removal test. We chose to use groups of 16 mice. Mice were trained for each task for 3 days before stroke surgery. We did not exclude any mice from the studies.

#### Accelerating rotarod test

Mice were placed on an accelerating rotarod apparatus (MK-610, Muromachi Kikai, Japan), in which the speed increased from 0 to 40 rpm over 4 min. The time that mice could remain on the rotating cylinder was measured. Three trials were performed, and the best latency to fall was used for the analysis.

#### Adhesive removal test

Mice were placed in the testing box. An adhesive tape (0.3 × 0.4 cm) was applied to the contralesional forepaw. The time to remove the tape from the paw was recorded. Each mice received a single trial.

### Administration of KUS121

KUS121 was dissolved in 5% cremophor EL (Sigma) in PBS to make a 20 μg/μL solution. In the focal ischemia stroke mouse model, KUS121 was injected intravenously via the tail vein or intraperitoneally at 100 mg/kg or 50 mg/kg, respectively.

### Statistical analysis

All values are expressed as means ± standard error (SE) unless stated otherwise. Differences with a probability value of *p* < 0.05 were considered to be statistically significant.

### Ethical approval

All procedures were performed in accordance with the guidelines for animal experimentation from the ethical committee of Kyoto University. All experimental protocols were approved by the animal experimentation committee of Kyoto University.

## Supplementary information

Supplementary information
