# Supplementary material for: KUS121, a valosin-containing protein modulator, attenuates ischemic stroke via preventing ATP depletion
Source: Sci Rep. 2019 Aug 8;9:11519. doi: 10.1038/s41598-019-47993-w (PMC6687885; doi:10.1038/s41598-019-47993-w)

Supplementary information

KUS121, a valosin-containing protein modulator, attenuates ischemic stroke via preventing ATP depletion

Hisanori Kinoshita[1], Takakuni Maki[1*], Ken Yasuda[1], Natsue Kishida[2], Norio Sasaoka[3], Yasushi Takagi[2], Akira Kakizuka[3]*, Ryosuke Takahashi[1]

[1] Department of Neurology, Kyoto University Graduate School of Medicine

[2] Department of Neurosurgery, Kyoto University Graduate School of Medicine

[3] Laboratory of Functional Biology, Kyoto University Graduate School of Biostudies

* Corresponding author: Takakuni Maki and Akira Kakizuka

Lead contact: Takakuni Maki, Department of Neurology, Kyoto University Graduate School of Medicine, 54 Shogoin-Kawahara-cho, Sakyo-ku, Kyoto, 606-8507 Japan, TEL; +81-75-751-3766, FAX; +81-75-751-9416, E-mail: harutoma@kuhp.kyoto-u.ac.jp

<u>Inventory of Supplementary Information</u>

Supplementary Figure S1-4 and figure legends

Supplementary figure legends

Supplementary Figure S1

(a) Western blot analyses of rat primary cortical neuron and oligodendrocyte lysates for Neuronal Nuclei (NeuN) and myelin basic protein (MBP). (b) Left: microtubule-associated protein 2 (MAP2) stained rat primary cortical neurons. Right: MBP stained rat primary cortical oligodendrocyte. Scale bar: 50 μm. (c) Western blot analyses of cerebral cortex lysates for Neurofilament heavy polypeptide (NF-H) and Glutathione S-transferase pi (GST-π). The cerebral cortex of B-17 mice was collected 24 h after transient occlusion of the distal MCA. The ratio of NF-H or GST-π to β-actin expression levels was calculated ($n = 4$; *, $p < 0.05$).

Supplementary Figure S2

(a) Cell viability measured by WST (water-soluble tetrazolium salt) assay. Rat primary cortical neurons were exposed to oxygen glucose deprivation (OGD) for 2 h, 4 h or 6 h. (b) Rotarod retention time and (c) the time to remove an adhesive tape of C57BL/6 mice subjected to transient occlusion of the distal portion of left middle cerebral artery (MCA). Neurological function was measured by the accelerating rotarod apparatus and adhesive removal test 1 d, 2 d, 3 d and 7 d after occlusion of the distal MCA ($n = 16$).

Supplementary Figure S3

Original Western blots of trimmed panels in Figure 2b (a), Figure 3e (b), Figure 4a (c).

Supplementary Figure S4

Original Western blots of trimmed panels in Supplementary Figure S1a (a), Supplementary Figure S1c (b).

Supplementary Figure S1

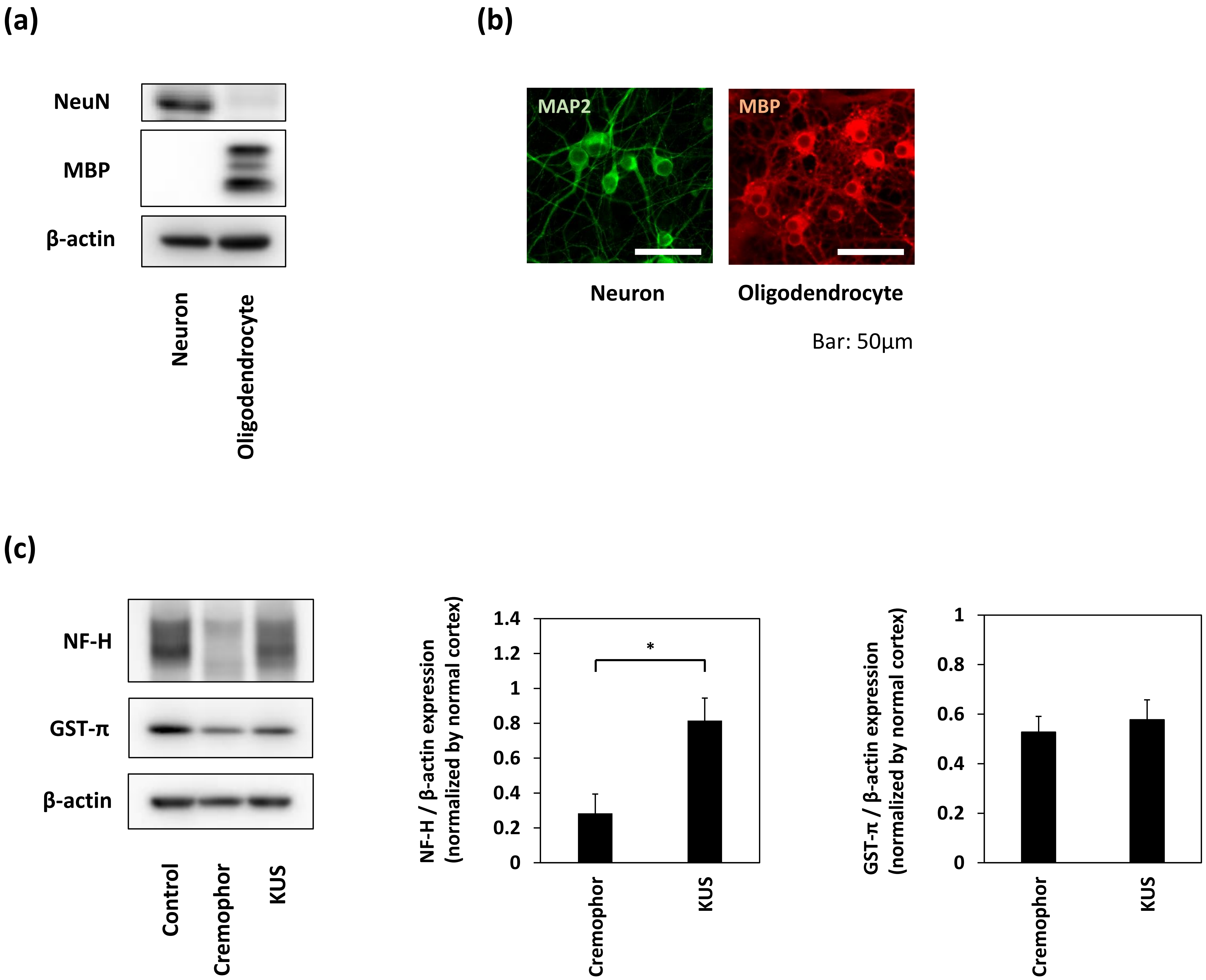

Supplementary Figure S2

(a)

(b)

(c)

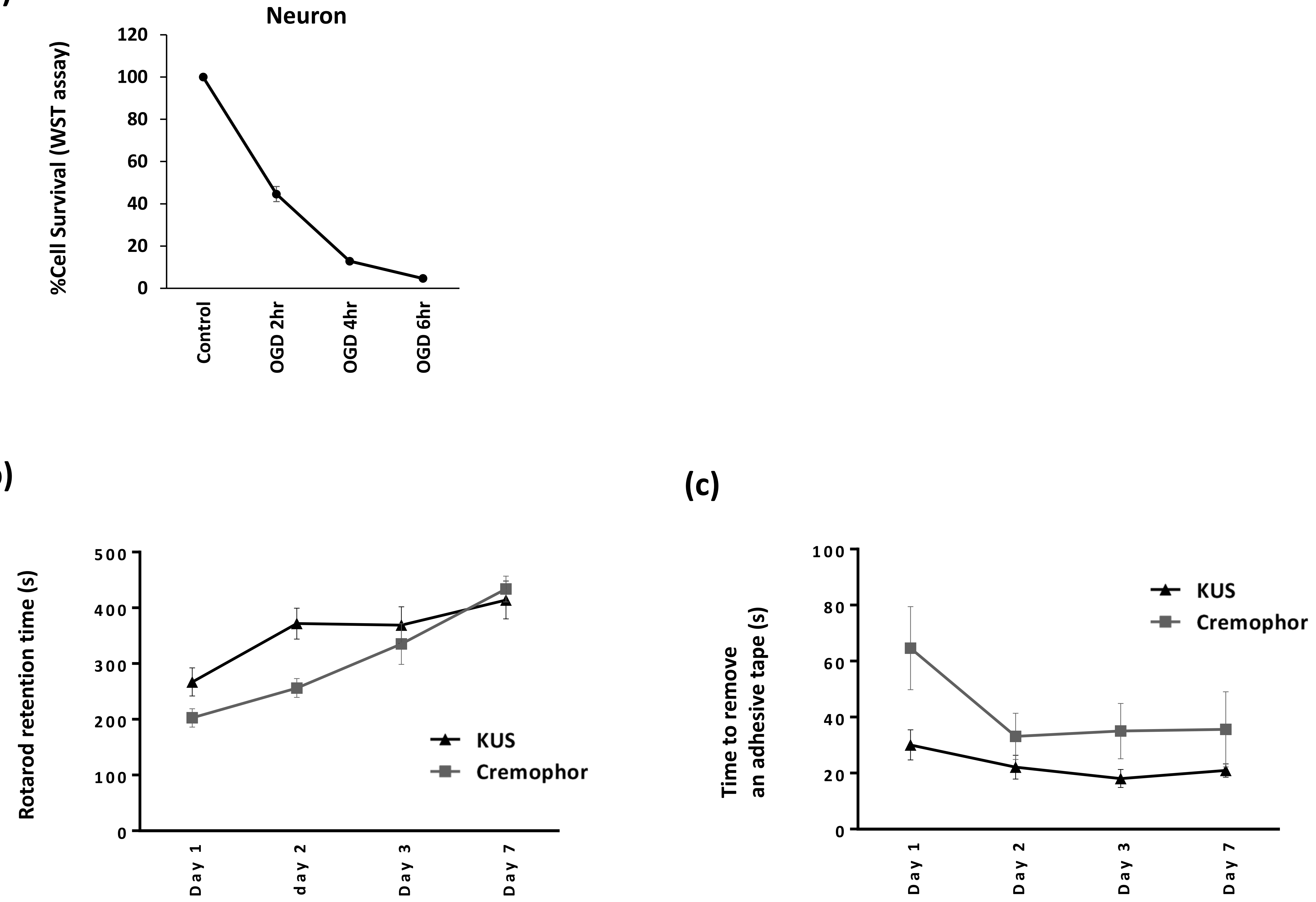

Supplementary Figure S3

(a) Figure 2b

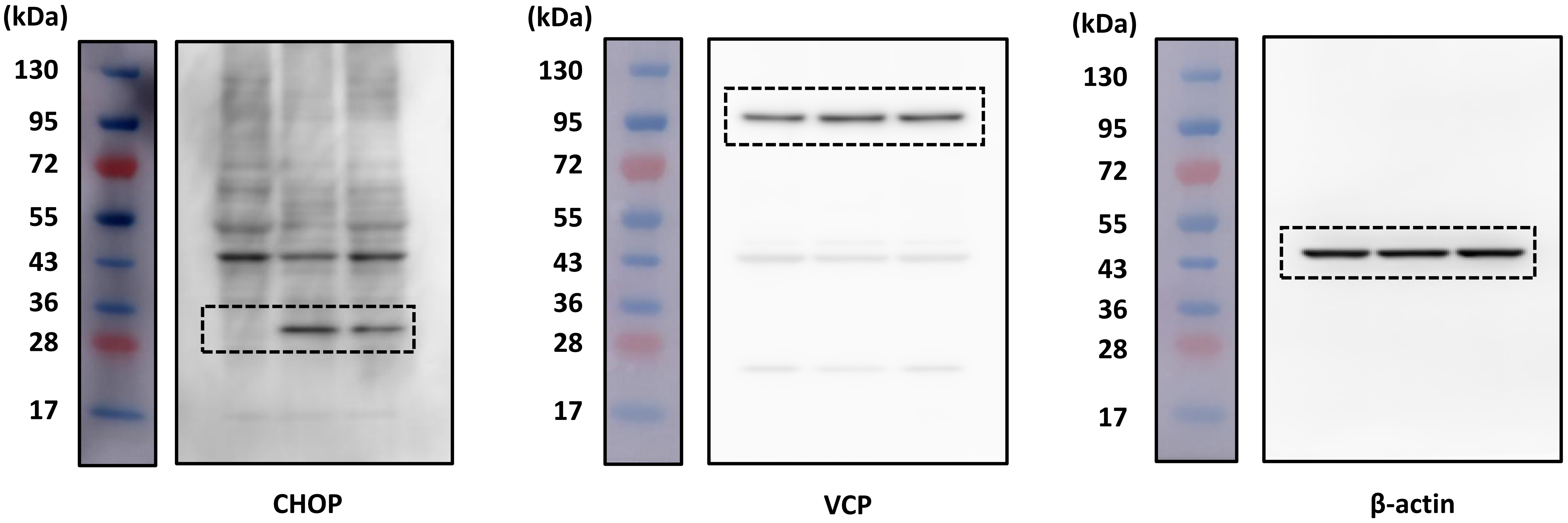

(b) Figure 3e

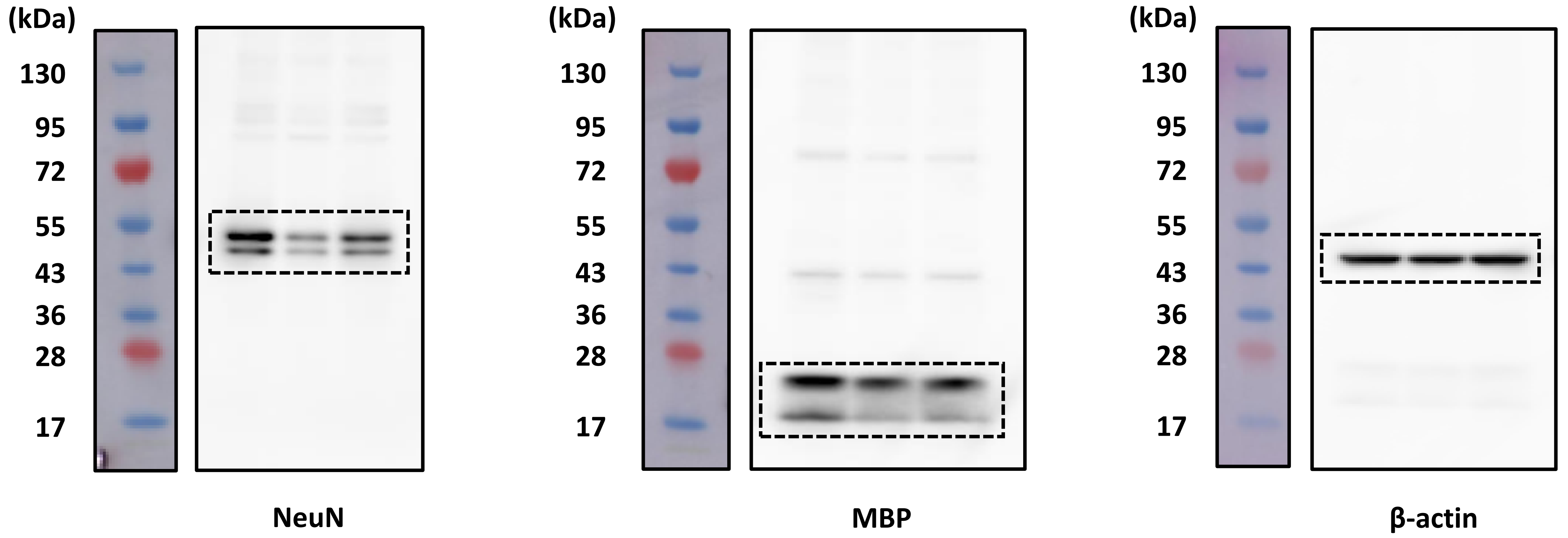

(c) Figure 4a

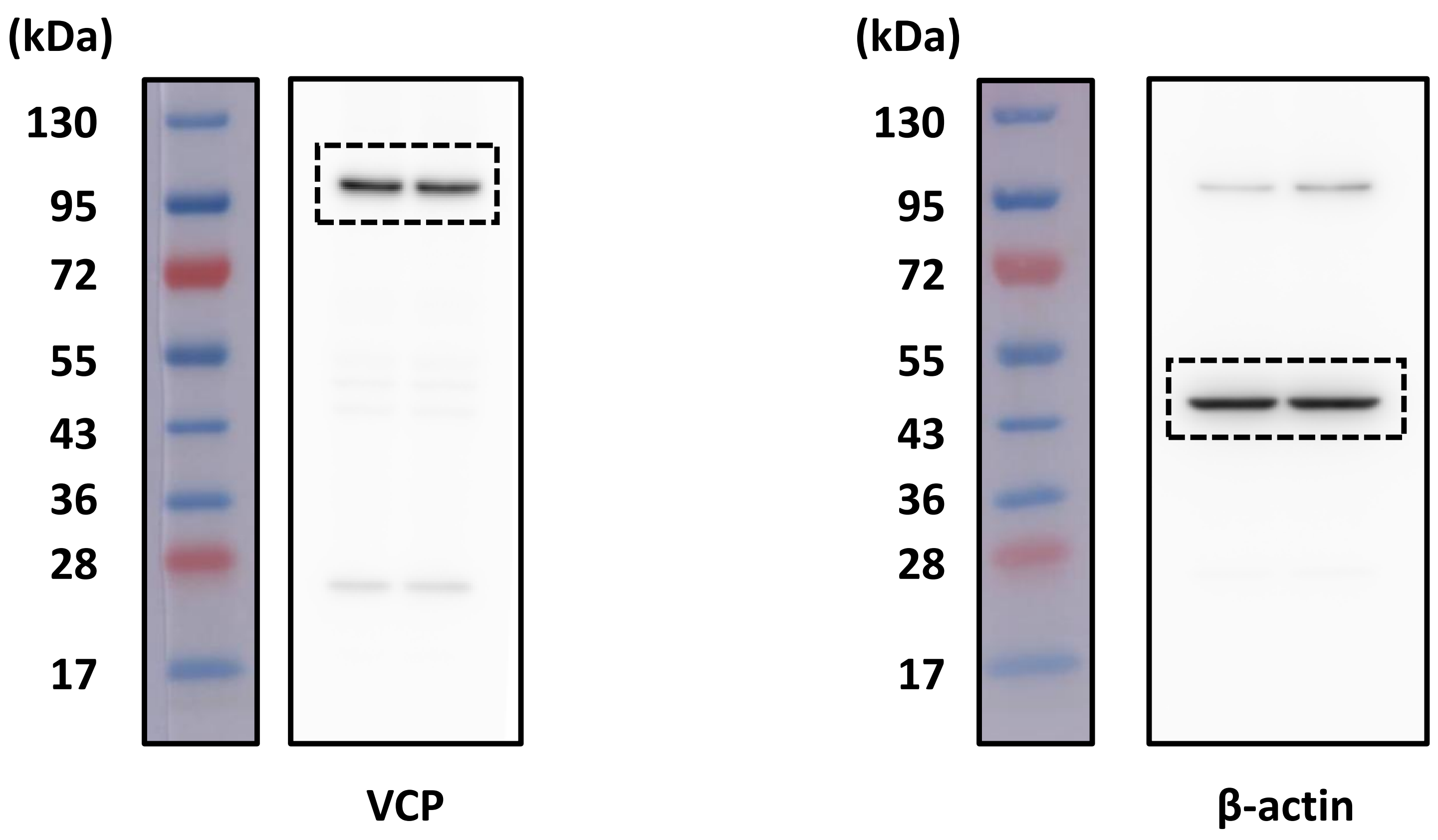

Supplementary Figure S4

(a) Supplementary Figure S1a

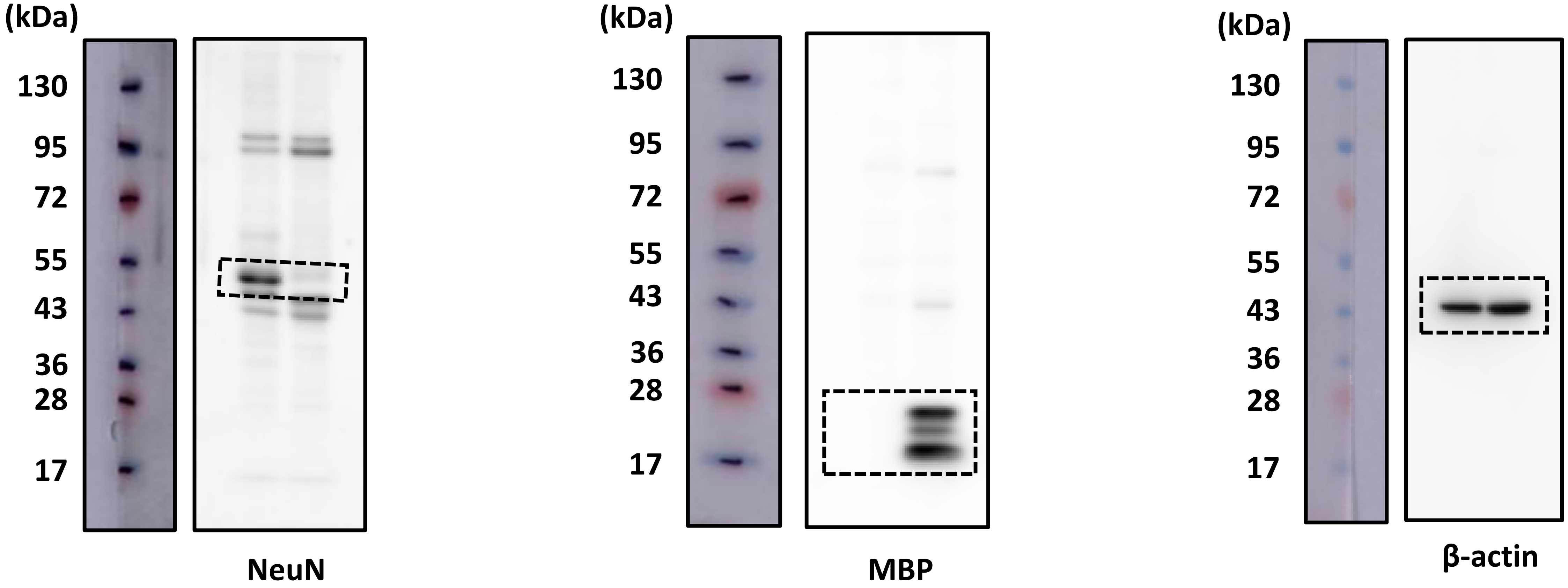

(b) Supplementary Figure S1c

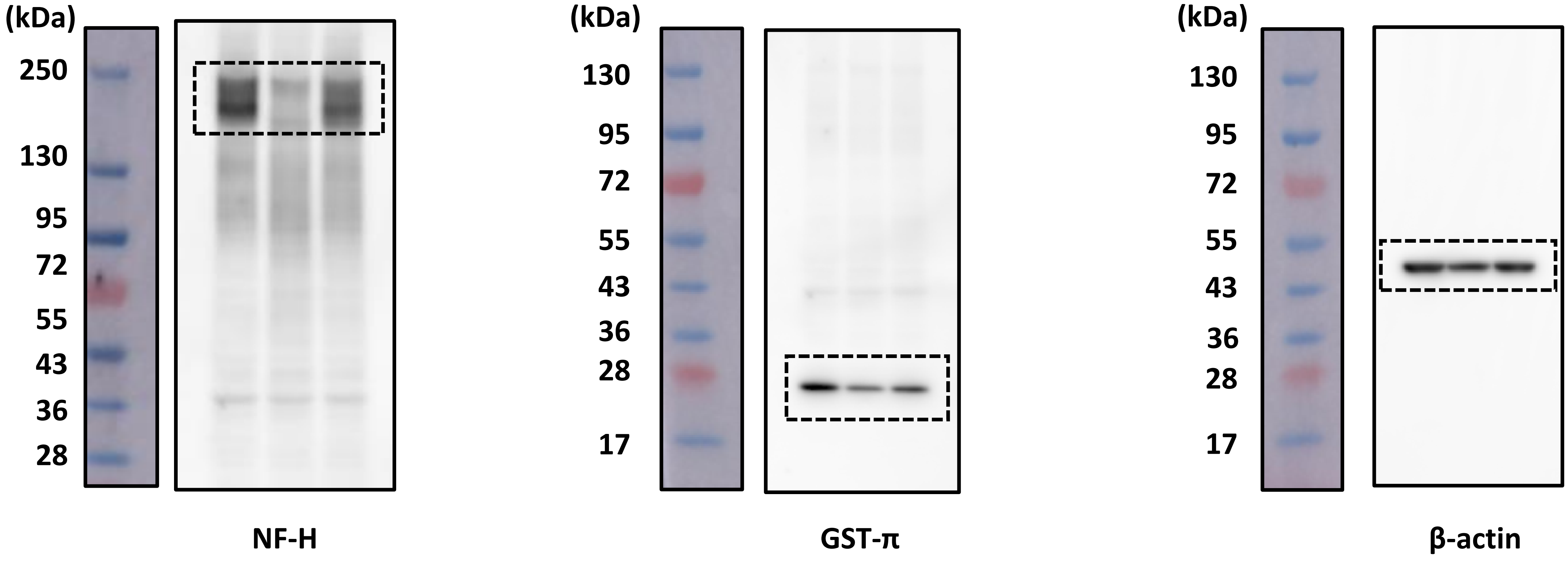

Supplement: Supplementary file 1 — Supplementary information [file 41598_2019_47993_MOESM1_ESM.pdf]
